# Impact of the Kaiser score on clinical decision-making in BI-RADS 4 mammographic calcifications examined with breast MRI

**DOI:** 10.1007/s00330-019-06444-w

**Published:** 2019-12-03

**Authors:** G. J. Wengert, F. Pipan, J. Almohanna, H. Bickel, S. Polanec, P. Kapetas, P. Clauser, K. Pinker, T. H. Helbich, P. A. T. Baltzer

**Affiliations:** 1 Department of Biomedical Imaging and Image-guided Therapy, Medical University of Vienna, Waehringer Guertel 18-20, Vienna, 1090 Austria; 2 Institute of Diagnostic Radiology, University of Udine, Udine, Italy; 3 Security Forces Hospital, Riyadh, Saudi Arabia; 4 Department of Radiology, Breast Imaging Service, Memorial Sloan Kettering Cancer Center, New York, NY USA

**Keywords:** Magnetic resonance imaging, Calcifications, Scoring system, Clinical decision-making, Breast cancer

## Abstract

**Objectives:**

To investigate whether the application of the Kaiser score for breast magnetic resonance imaging (MRI) might downgrade breast lesions that present as mammographic calcifications and avoid unnecessary breast biopsies

**Methods:**

This IRB-approved, retrospective, cross-sectional, single-center study included 167 consecutive patients with suspicious mammographic calcifications and histopathologically verified results. These patients underwent a pre-interventional breast MRI exam for further diagnostic assessment before vacuum-assisted stereotactic-guided biopsy (95 malignant and 72 benign lesions). Two breast radiologists with different levels of experience independently read all examinations using the Kaiser score, a machine learning–derived clinical decision-making tool that provides probabilities of malignancy by a formalized combination of diagnostic criteria. Diagnostic performance was assessed by receiver operating characteristics (ROC) analysis and inter-reader agreement by the calculation of Cohen’s kappa coefficients.

**Results:**

Application of the Kaiser score revealed a large area under the ROC curve (0.859–0.889). Rule-out criteria, with high sensitivity, were applied to mass and non-mass lesions alike. The rate of potentially avoidable breast biopsies ranged between 58.3 and 65.3%, with the lowest rate observed with the least experienced reader.

**Conclusions:**

Applying the Kaiser score to breast MRI allows stratifying the risk of breast cancer in lesions that present as suspicious calcifications on mammography and may thus avoid unnecessary breast biopsies.

**Key Points:**

*• The Kaiser score is a helpful clinical decision tool for distinguishing malignant from benign breast lesions that present as calcifications on mammography.*

*• Application of the Kaiser score may obviate 58.3–65.3% of unnecessary stereotactic biopsies of suspicious calcifications.*

*• High Kaiser scores predict breast cancer with high specificity, aiding clinical decision-making with regard to re-biopsy in case of negative results.*

## Introduction

Calcifications detected on mammography are found in almost one-third of patients screened for breast cancer [1]. While mammographic calcifications are considered an early indicator of breast cancer, in particular DCIS (ductal carcinoma in situ), not all biopsies yield malignant results. Therefore, mammographic calcifications regularly require further diagnostic workup with stereotactic-guided biopsy, a significant proportion of which yield benign findings, and could thus potentially be avoided [2–4]. A systematic review and meta-analysis reported varying malignancy rates in mammographic calcifications ranging between 6 and 82% depending on their mammographic appearance. Interestingly, the paper reveals that malignancy rates in all BI-RADS feature combinations exceed BI-RADS 3 benchmarks [5]. Therefore, invasive workup rather than short-term follow-up is formally required. Second, due to the preferred use of one-way vacuum-biopsy needles and marker placement for follow-up or surgery, stereotactic biopsy is costlier than ultrasound-guided biopsies. One potential way to exclude malignancy in these cases, and thereby reduce the number of unnecessary biopsies, would be to use contrast-enhanced magnetic resonance imaging (MRI). However, while several reports have demonstrated a diagnostic role for breast MRI with regard to calcifications, its application in this setting has yielded variable results, and it is not yet recommended for this purpose [6–8]. One reason is the lack of definite diagnostic criteria that would enable objective and reliable exclusion of malignancy in case of lesions that present as mammographic calcifications. An interpretation of breast MRI using a clinical decision rule could alleviate this issue. The *Kaiser score* is such a decision-making tool and has demonstrated its usefulness in downgrading MRI-detected suspicious breast lesions through a formalized combination of the BI-RADS lexicon descriptors [9–11].

Consequently, we investigated the diagnostic performance and inter-reader agreement of the Kaiser score in the diagnostic workup of mammographic calcifications by breast MRI to potentially avoid unnecessary breast biopsies.

## Materials and methods

### Study design and patient population/study cohort

Our local institutional review board approved this retrospective, cross-sectional, single-center study, and waived the necessity for written informed consent. A search of eligible patients with suspicious calcifications on mammography, with no asymmetric densities, focal asymmetries, or mass lesions, and who were negative on sonography, and were undergoing further diagnostic workup with percutaneous breast biopsy or surgery was performed on the institutional database. This database search between October 2012 and December 2015 revealed 454 consecutive patients, of which 167 patients, with a mean age of 53 years (age range from 24 to 85 years), underwent breast MRI before biopsy. Referral to MRI largely depended on the availability of examination time slots and was mainly used to plan the biopsy in cases of not well-circumscribed or multiple calcifications covering a discontinuous area. All calcifications in the database were initially classified as BI-RADS 4 based on BI-RADS lexicon criteria. As the database cases were diagnosed during assessment, breast cancer screening, and curative mammography, no subdivision into BI-RADS 4 a–c categories was done. No high-risk screening examinations were included in this study.

### Imaging

Full-field digital mammography was performed on a standard device at our institute (Mammomat Inspiration, Siemens Healthineers) or on other standard devices at other affiliated facilities (Selenia Dimensions, Hologic, or Senographe Essential Mammography, GE Healthcare) with standard craniocaudal and mediolateral–oblique views. Lateromedial or magnification views were done in a proportion of the investigated patients on the interventional breast radiologists’ discretion but were never used to downgrade BI-RADS 4 findings.

Diagnostic MRI examinations and protocols were performed for each woman, in the prone position, using a dedicated breast coil on different referring units on 1.5-T (MAGNETOM AvantoFit, Siemens Healthineers with a dedicated 18-channel breast coil, Noras) and 3.0-T machines (MAGNETOM TimTrio, Siemens Healthineers with a 4-channel breast coil, In Vivo; and MAGNETOM PrismaFit, Siemens Healthineers with a dedicated 16-channel breast coil, Sentinel) in accordance with the EUSOMA (European Society of Breast Cancer Specialists) and EUSOBI (European Society of Breast Imaging) recommendations [12, 13].

### Data analysis

All MR examinations were evaluated by two independent, fellowship-trained breast radiologists (R1, 7 years of experience; and R2, 8 years of experience). The readers were blinded of histopathological results.

**Fig. 1:**
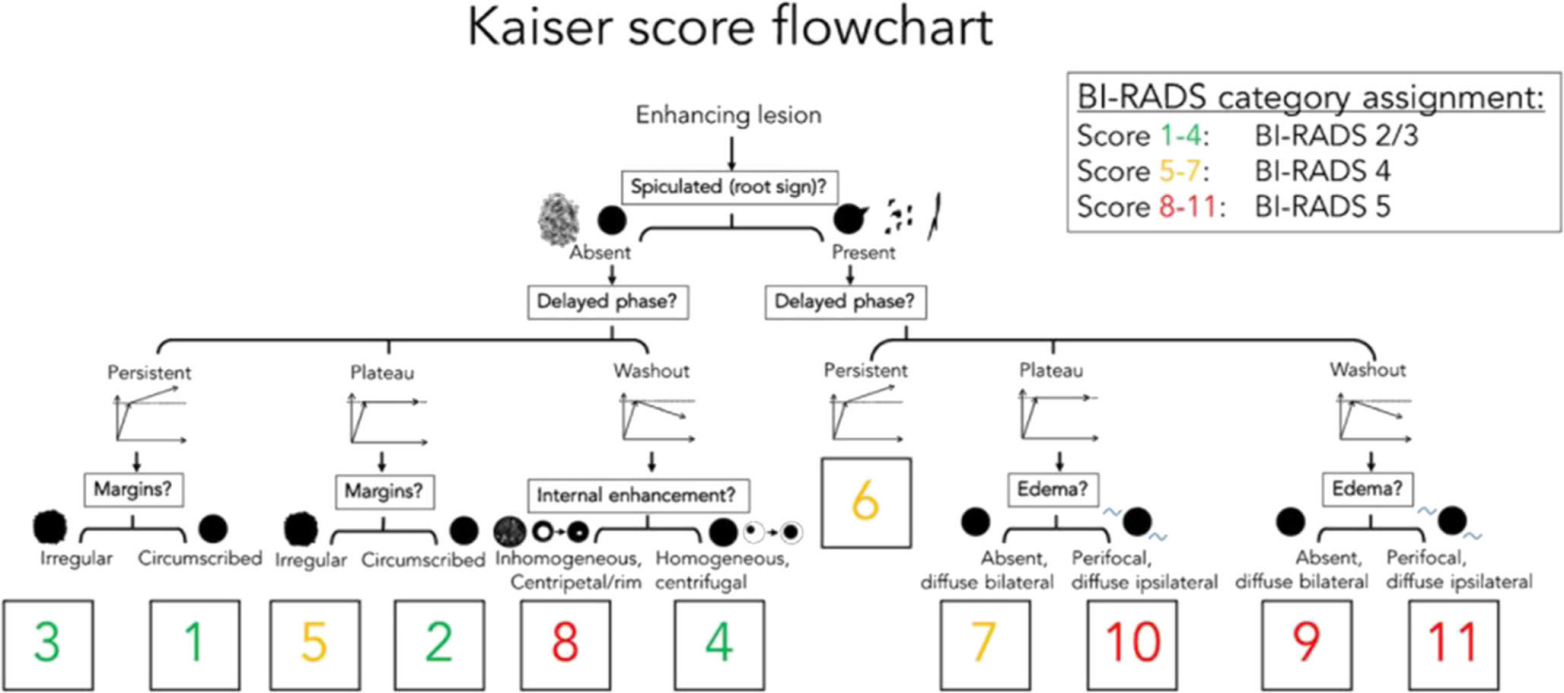
The Kaiser score flowchart modified after [9]. This illustration first described by Baltzer and coworkers in 2013 [10] is an intuitive tool that is followed from the top to the bottom and assigns four diagnostic categories that help the reader determine the likelihood of a benign or malignant finding (1 = lowest, 11 = highest). The outcome can be assigned to the appropriate BI-RADS category

The readers were asked to apply a clinical scoring system (the Kaiser score, formerly referred to as the *Tree* flowchart) that is composed of five BI-RADS-derived diagnostic criteria and contains 11 assignment categories which correspond to an increased probability of malignancy ranging from 1 to 11 (Fig. 1) [9]. This algorithm can also be accessed online: http://www.meduniwien.ac.at/kaiser-score/.

Following the self-explanatory flowchart with the support of sample images, each reader had to assign a diagnostic category for each lesion.

### Reference standard

Histopathological diagnosis was regarded as the reference standard, and was analyzed by board-certified and experienced breast pathologists through percutaneous, console-based, stereotactic-guided, vacuum-assisted breast biopsy using an ATEC® System (Hologic) with a 9G needle using aseptic techniques or using open surgery. Lesions of uncertain malignant potential (B3) underwent surgery after wire localization and the final histopathological diagnosis was used for further analysis. As is standard in our institution, all patients with negative biopsy results underwent annual follow-up for 2 years and afterwards returned to bi-annual screening. Patients with breast cancer underwent intensified clinical and imaging follow-up. A follow-up of at least 2 years was available in all patients.

### Statistical analysis

Statistical analysis was performed using the Statistical Package for the Social Sciences (IBM SPSS Statistics version 23.0) and MedCalc statistical software, version 15.4 for Windows. All calculations were performed on a per-lesion basis. Final histopathological diagnosis, which was regarded as the reference standard for each lesion, was obtained from reports prospectively stored at our institutional database and which was prospectively checked for congruence during interdisciplinary meetings on a weekly basis. A receiver operating characteristic (ROC) analysis was performed to establish the complete diagnostic performance of the Kaiser score, measured by the area under the ROC curve (AUC). In addition, sensitivity, specificity, and likelihood ratios were calculated on the basis of the 11 assignment categories, at cutoff values of > 4, which indicated malignancy. Inter-reader agreement of the dichotomized Kaiser score categories (positive > 4 vs negative ≤ 4) was analyzed using a Cohen’s kappa coefficient. The strength of agreement was expressed in *k* values. Sensitivity and specificity differences between readers were probed by McNemar tests for paired proportions. A *p* value equal to or below 0.05 was considered statistically significant.

## Results

The final study cohort comprised 167 patients (age range, 24–85 years; mean age, 53 years) and 167 lesions. Median sizes in mammography (18, range 4–72 mm) and MRI (19, range 5–84 mm) did not differ (*p* > 0.05). Histopathology revealed 95 malignant and 72 benign lesions, resulting in a prevalence of malignancy of 56.9% (95% CI 49.3–64.2%). Fifty-one of 167 lesions presented as masses (30.5%), 29 (56.9%) of which were malignant, whereas 116 of 167 lesions (69.5%) presented as a non-mass enhancement, 66 (57.9%) of which were malignant. Of the 95 malignant lesions, 48 (50.5%) were pure DCIS and 47 (49.5%) were invasive cancers upon histopathology. The invasive cancers consisted of 40 invasive ductal carcinomas, five invasive lobular carcinomas, and two invasive tubular cancers. Thirty-three of the 47 invasive carcinomas (70.2%) had a DCIS component.

### Diagnostic performance and potential to avoid unnecessary breast biopsies

**Fig. 2:**
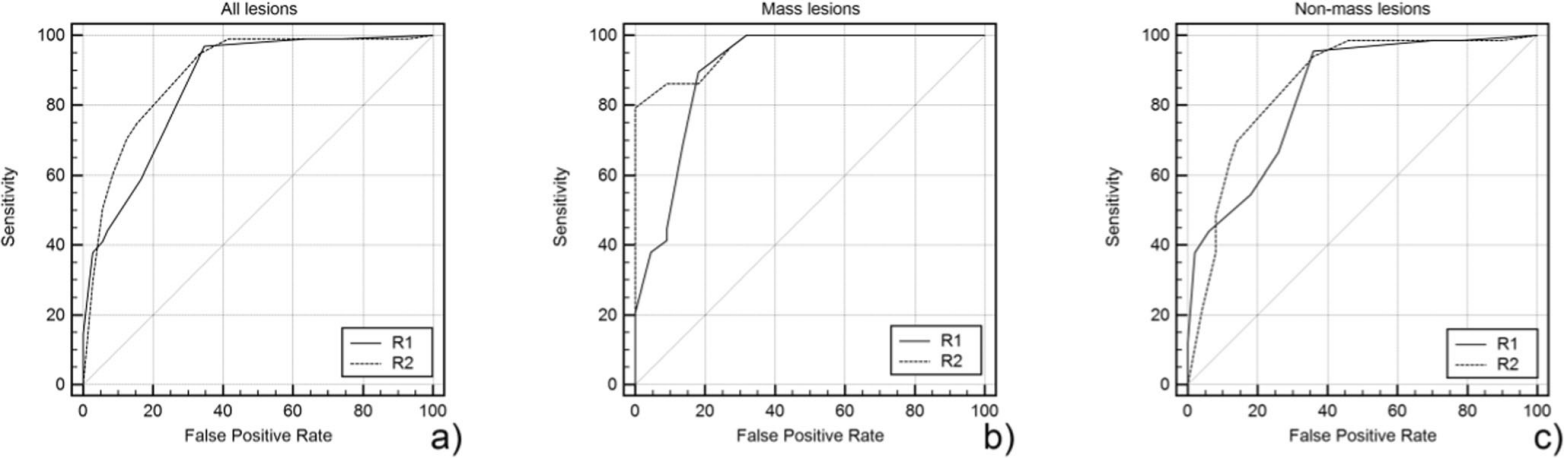
Receiver operating characteristics (ROC) curves of Kaiser score ratings for all (**a**), mass (**b**), and non-mass (**c**) lesions. Note that a high sensitivity level with a rule-out criterion (Kaiser score ≤ 4) was found in both readers (R1, R2). Lower false-positive rates were indicative for biopsy rate reduction

The AUC for all lesion diagnoses ranged between 0.859 and 0.889 for the Kaiser score. Regarding mass lesions, the AUC ranged between 0.904 and 0.963, and for non-mass lesions, the AUC ranged between 0.837 and 0.861 (Fig. 2, Table 1).

**Table 1:** Area under the ROC curves (ROC) for the Kaiser score (KS) reading for all three readers, all lesions, and separated by mass and non-mass lesions with their corresponding standard errors and 95% confidence intervals (CI)

	Area	95% CI	
		Lower Bound	Upper Bound
All lesions			
KS R1	0.859	0.797	0.908
KS R2	0.889	0.831	0.933
Mass lesions			
KS R1	0.904	0.789	0.969
KS R2	0.963	0.869	0.996
Non-mass lesions			
KS R1	0.837	0.757	0.899
KS R2	0.861	0.784	0.918

The Kaiser score achieved high sensitivity, ranging between 96.8% (92/95, two false-negative (FN), one DCIS G2 and G3 each, and one T1a invasive ductal cancer G2; all hormone receptor–positive in perimenopausal women aged 52, 54, and 58 years; lesion sizes 8, 7, and 5 mm) and 98.9% (94/95, one 8-mm FN DCIS G2, hormonal receptor–positive in a 52-year-old patient), with no significant difference between both readers or based on lesion presentation as a mass or non-mass (*p* > 0.05, respectively, Table 2). Specificity ranged between 58.3% (41/72) and 65.3% (46/72). As all investigated cases were rated BI-RADS 4 upon mammography and subsequently biopsied, these numbers reflect the rate of unnecessary biopsies that could have been avoided. The detailed results using the Kaiser score for lesion characterization, with the corresponding sensitivity and specificity values and 95% confidence intervals for each reader, are illustrated for mass and non-mass lesions in Table 2.

**Table 2:** Diagnostic parameters of the Kaiser score (KS) readings for all three readers stratified by lesion presentation as all lesions, mass lesions, or non-mass lesions

	Sensitivity (TP/TP + TN)	95% CI	Specificity (TN/TN + FP)	95% CI	+LR	−LR
All lesions (*n* = 167)						
KS R1	96.8 (92/95)	91.0–99.3	65.3 (46/72)	53.1–76.1	2.79	0.048
KS R2	98.9 (94/95)	94.3–100.0	58.3 (41/72)	46.1–69.8	2.37	0.018
Mass lesions (*n* = 51)						
KS R1	100.0 (29/29)	88.1–100.0	68.2 (15/22)	45.1–86.1	3.14	n.a.
KS R2	100.0 (29/29)	88.1–100.0	68.2 (15/22)	45.1–86.1	3.14	n.a.
Non-mass lesions (*n* = 116)						
KS R1	95.5 (63/66)	87.3–99.1	64.0 (32/50)	49.2–77.1	2.65	0.071
KS R2	98.5 (65/66)	91.8–100.0	54.0 (27/50)	39.3–68.2	2.14	0.028

**Fig. 3:**
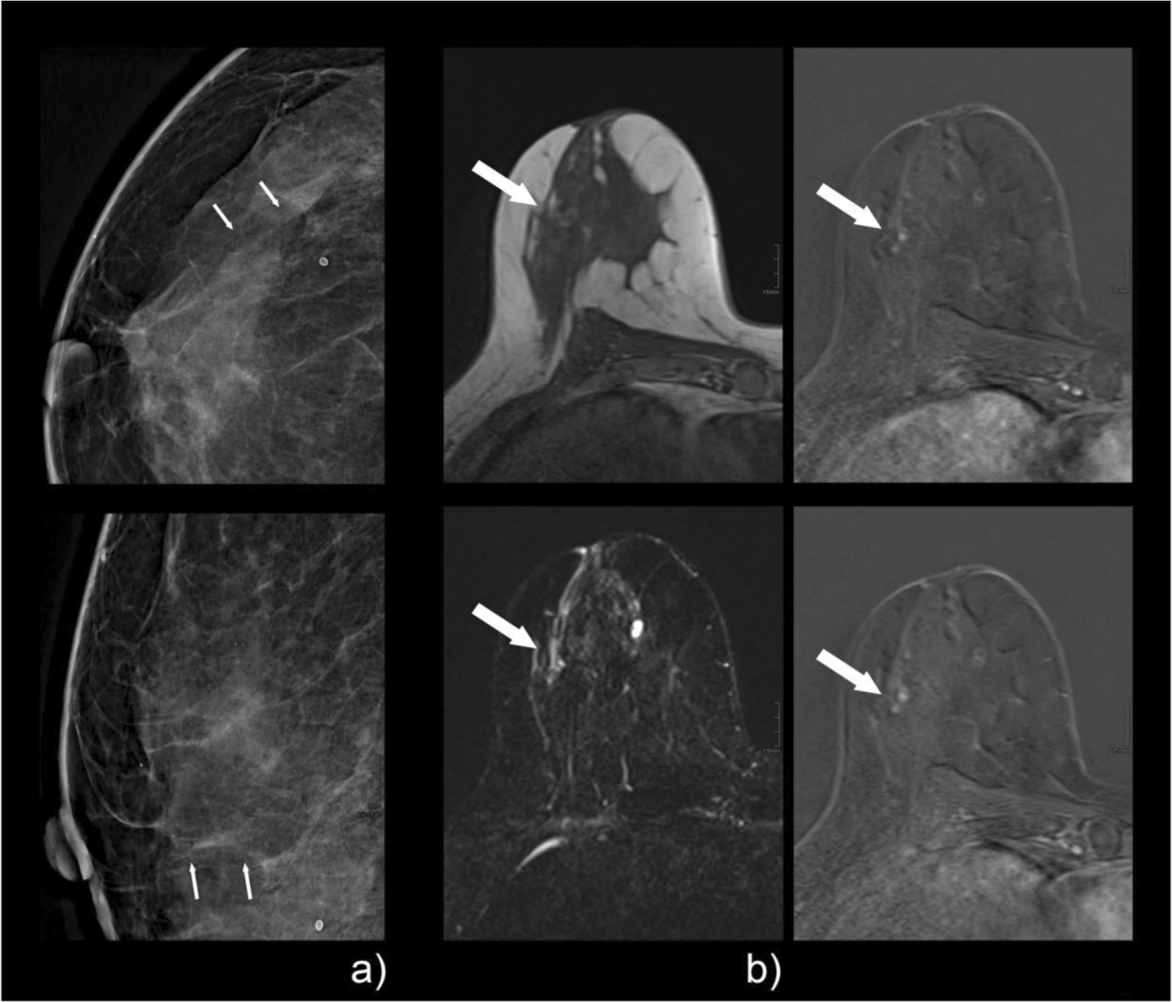
A 47-year-old patient with screen-detected amorphous calcifications with a linear distribution on mammography, classified as BI-RADS 4a (**a**, tiny arrows). The pre-interventional breast MRI showed a corresponding clumped non-mass enhancement with a linear distribution in the lateral quadrant of the right breast (**b**, large arrow). Margins are rather circumscribed without spiculations, and the curve type is persistent (**b**: upper right, early enhanced; lower right, late enhanced subtraction), which resulted in a Kaiser score of 1. If margins were rated as non-circumscribed, as was done by one reader, the resulting Kaiser score would have been 3, which would still have led to a BI-RADS 2/3 category assignment. Histopathologic workup of the stereotactic biopsy specimen revealed apocrine metaplasia with a small cholesterol granuloma: B2

**Fig. 4:**
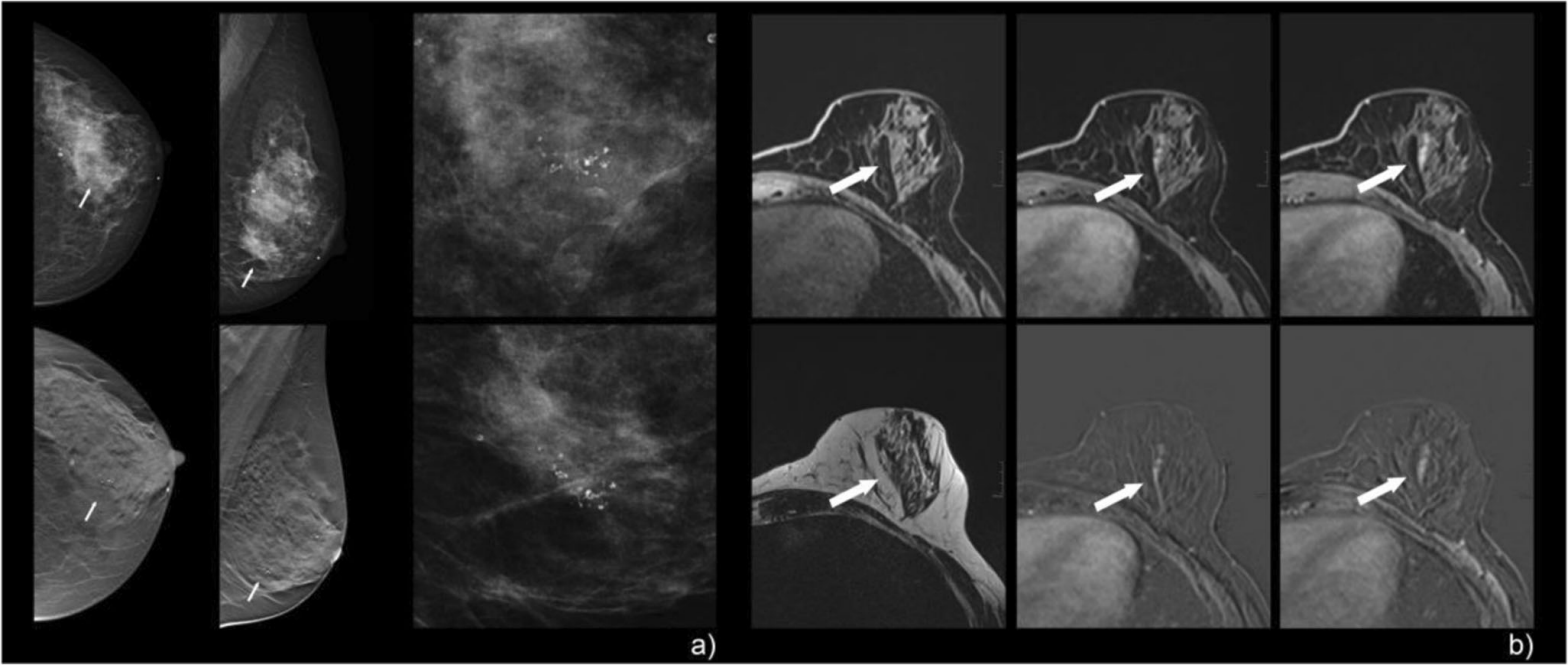
A 49-year-old woman with screen-detected grouped coarse heterogeneous calcifications (**a**, tiny arrows) on mammography/tomosynthesis, which was classified as BI-RADS 4. Pre-interventional breast MRI (**b**) shows a clumped, non-mass enhancement (large arrows) in a linear/segmental distribution, with a plateau curve in the nodular aspects (**b**: upper row from left to right shows pre-contrast, early, and late enhanced images, and the lower row shows T2w, early, and late subtractions). The finding was read as a Kaiser score of 5. Histopathology revealed an invasive ductal carcinoma: B5a

**Fig. 5:**
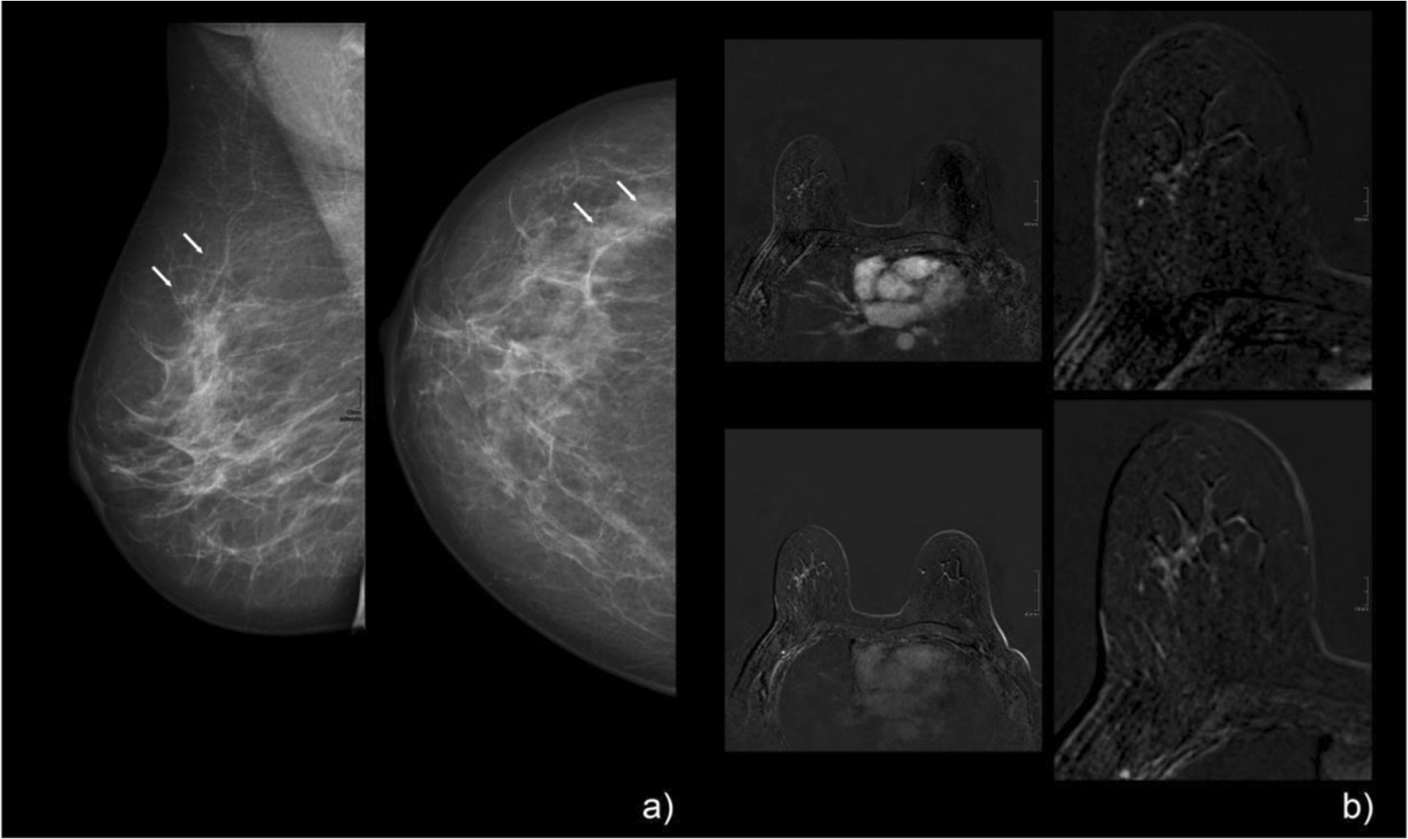
A 53-year-old woman with screen-detected calcifications in the right breast. Mammographic CC and MLO views (**a**) showed grouped, fine pleomorphic calcifications that were assigned BI-RADS 4b. Pre-interventional breast MRI (**b**) demonstrated a corresponding regional, heterogeneous, enhancing non-mass lesion in the right breast. Persistent enhancement kinetics (**b**: upper right, early enhanced; lower right, late enhanced subtraction) and irregular margins resulted in a Kaiser score of 3 (corresponding to BI-RADS 2/3, benign finding). Stereotactic biopsy revealed non-specific proliferative changes in the tumor-free breast parenchyma with calcifications: B2. MRI follow-up (not shown here) showed no suspicious findings

**Fig. 6:**
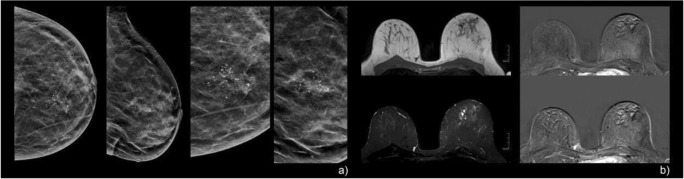
A 52-year-old woman with screen-detected, regionally distributed, coarse heterogeneous calcifications in the left breast (**a**). Pre-interventional breast MRI revealed no significant enhancement, although two readers interpreted the finding as a heterogeneous non-mass enhancement in a segmental to regional distribution, with a persistent curve type and non-circumscribed margins (**b**: the left column illustrates the corresponding T1w, top, and STIR, bottom, as well as early and late enhanced subtractions, respectively). Further, segmentally arranged cystic structures were evident. The finding was read as a Kaiser score of 3 (BI-RADS 2/3). Stereotactic biopsy revealed fibrocystic changes with small ductal epithelial proliferation without atypia and sparse calcifications: B2

Examples of mammographic calcifications and corresponding MRI images, including the Kaiser score evaluation, are given in Figs. 3, 4, 5, and 6.

### Inter-reader agreement

The Cohen kappa agreement among the two readers for the differentiation of malignant from benign breast lesions was moderate, with *k* = 0.510 (95% CI 0.365–0.655).

## Discussion

The results of our study highlight the diagnostic potential of applying the Kaiser score to breast MRI in the diagnostic workup of suspicious mammographic calcifications. This clinical decision tool, which guides the reader through an intuitive, stepwise decision tree, allows a distinction of the underlying pathology by means of objective criteria. According to our results, the Kaiser score largely allows the exclusion of clinically significant breast cancer and, thus, obviates the need for unnecessary invasive procedures, with an effective reduction of up to 65.3% of unnecessary stereotactic breast biopsies.

The Kaiser score, named after Werner Alois Kaiser, a German radiologist and pioneer in clinical breast MRI, has shown substantial usefulness in the completion of lesion descriptors of the BI-RADS lexicon to reliably differentiate malignant from benign lesions on dynamic contrast-enhanced breast MRI [9–11]. According to the Kaiser score, the probability of a lesion being malignant can range from 1 (minimal) to 11 (maximal). Our findings revealed that Kaiser scores ≤ 4 largely exclude malignancy and yielded a low number of false-negative findings, i.e., all but one non-invasive and hormonal receptor–positive tumor. Our findings were applicable to mass and non-mass lesions alike, thereby stressing the applicability of the Kaiser score in the investigated population. The Kaiser score may thus be used to avoid further invasive diagnostic approaches, such as percutaneous breast biopsies or open surgical procedures. In addition to the potential to avoid biopsies, MRI might also be helpful after inconclusive negative biopsy results. As higher Kaiser scores associate with high malignancy rates, a positive MRI might indicate the need to repeat a negative biopsy. This provides the opportunity to stratify women with suspicious mammographic calcifications into those who require or do not require further diagnostic workup, and has, therefore, the economic potential to reduce stereotactic biopsy or surgery time slots, and, ultimately, healthcare costs. Although, due to inconsistent healthcare policies in Europe, definitive numbers are difficult to provide, a diagnostic MRI scan is usually cheaper and may be less time-consuming compared with stereotactic breast biopsies, though procedure times vary depending on experience and can be substantially reduced using more modern equipment such as tomosynthesis guidance [14]. The relation of these costs currently is 2:1 in our country, as costs vary between countries and settings (academic, public, private); thus, a dedicated analysis regarding the cost effectiveness of using MRI before stereotactic biopsy of mammographic calcifications is not possible at this point. In addition, while MRI is not an invasive procedure such as stereotactic biopsy, the potential risks of contrast medium application need to be considered [15, 16]. The main potential disadvantage of MRI, however, is waiting time in case of limited availability, leading to stress and anxiety in patients. In this respect, contrast-enhanced mammography is receiving increased attention as it promises similar diagnostic information as MRI without the need to re-schedule the patient, thus avoiding a diagnostic delay [17]. Contrast-enhanced mammography, however, increases the patient’s exposure to ionizing radiation and does also require the application of contrast media associated with higher rates of adverse effects compared with MRI contrast media. It should also be stressed that this rather novel method has not been extensively tested regarding the sensitivity for non-invasive cancer and non-mass enhancements.

Since its introduction, the Kaiser score has been applied in a limited number of clinical settings [10]. Marino et al demonstrated the applicability of the Kaiser score in a problem-solving setting [11]. They found a higher inter-reader agreement when using the Kaiser score compared with BI-RADS. Further, the authors showed an improved diagnostic performance for inexperienced readers. Another study by Woitek et al demonstrated the potential to avoid unnecessary MRI-guided biopsies in MRI-only lesions using the Kaiser score. Using a cutoff value ≤ 2, more than 25% of the false-positive biopsies could have been correctly classified as benign without yielding false-negative cases [18]. The authors further demonstrated a consistently high diagnostic accuracy and inter-reader agreement for the Kaiser score, even though their diagnostic MRI scans came from multiple units and field strengths. This may be one explanation why their cutoff for malignancy was lower compared with ours, and to the results of Marino et al. Our study adds to the body of evidence and also suggests a role for the Kaiser score in the emerging indication of resolving mammographic calcifications. A recent meta-analysis demonstrated the potential of contrast-enhanced breast MRI to downgrade BI-RADS 4 calcifications while pointing out that there are no established diagnostic criteria in this setting [19]. According to our results, the Kaiser score fills this gap.

Some limitations of the present study should be mentioned. First, our study was retrospective; thus, though we could demonstrate the potential of the Kaiser Score to diagnose breast MRI lesions found in patients with suspicious mammographic calcifications, we did not prospectively demonstrate the potential to avoid unnecessary biopsies. The rather high prevalence of malignancy that was higher than the general prevalence of malignancy in mammographic calcifications suggests a certain selection bias toward more suspicious cases [5]. The effect of this potential selection bias due to time slot availability cannot be confirmed or excluded based on the retrospective nature of our study. Potential effect is overestimation of sensitivity while underestimating specificity. Therefore, prospective studies are needed to both confirm the potential of breast MRI and the Kaiser score in this setting. In addition, the analysis of the inter-reader agreement to differentiate benign from malignant breast lesions revealed a moderate result, indicating that diagnosis of lesions presenting as mammographic calcifications is challenging on MRI, even when using a standardized diagnostic algorithm. It should be noted that morphological interpretation of MRI images is always a subjective task—this is why conventional BI-RADS feature interpretation has a limited inter-reader agreement which has been reported as being lower compared with the Kaiser score [11]. However, considering the variations in sensitivity and specificity observed within this study, the results were fairly robust and thus encouraging in the setting of a breast MRI workup for suspicious mammographic calcifications. In addition, the analyzed images came from several different MRI units and protocols. This should, however, not be considered a limitation, but rather, a strength of this study, as it reflects clinical practice in a tertiary care assessment center, and thus, confirms the applicability of our findings to clinical practice. Due to the heterogeneous protocols, we did not evaluate whether background parenchymal enhancement does influence the diagnostic certainty of the Kaiser score in the investigated setting: this remains an interesting research gap as the effect of background parenchymal enhancement on the diagnostic performance of breast MRI remains controversial [20, 21].

In conclusion, we demonstrated that the application of the Kaiser score in breast MRI can downgrade up to 65.3% of lesions that present as suspicious mammographic calcifications. This suggests the potential to avoid unnecessary stereotactic biopsies in a substantial proportion of cases.

## Abbreviations

AUC: Area under the ROC curve
BI-RADS: Breast Imaging-Reporting and Data System
CC: Craniocaudal view
CI: Confidence interval
DCIS: Ductal carcinoma in situ
EUSOBI: European Society of Breast Imaging
EUSOMA: European Society of Breast Cancer Specialists
FP: False positive
KS: Kaiser score
LR: Likelihood ratio
MLO: Mediolateral–oblique view
MRI: Magnetic resonance imaging
*n*: Number of cases
R1: Reader 1
R2: Reader 2
ROC: Receiver operating characteristics
STIR: Short tau inversion recovery sequence
T1: T1-weighted sequence
T2: T2-weighted sequence
TN: True negative
TP: True positive
